# SNORA73B promotes endometrial cancer progression through targeting *MIB1* and regulating host gene RCC1 alternative splicing

**DOI:** 10.1111/jcmm.17850

**Published:** 2023-07-24

**Authors:** Xi Chen, Qian‐hui Li, Bu‐min Xie, Yu‐meng Ji, Yang Han, Yang Zhao

**Affiliations:** ^1^ Department of Obstetrics and Gynecology, Department of Gynecologic Oncology Research Office, Key Laboratory for Major Obstetric Diseases of Guangdong Province The Third Affiliated Hospital of Guangzhou Medical University Guangzhou China

**Keywords:** alternative splicing, endometrial cancer, notch pathway, pseudouridine, SNORA73B, ubiquitin

## Abstract

Endometrial cancer (EC) is a common gynaecological malignant tumour with unclear pathogenesis. Small nucleolar RNA (snoRNA) is involved in many biological processes, including those of cancers. Using the Cancer Genome Atlas (TCGA) database, the expression pattern of a snoRNA, SNORA73B, was analysed. The biological functions of SNORA73B were assessed by in vitro proliferation, apoptosis, migration, and invasion assays and in vivo by the xenograft model. RNA sequencing (RNA‐seq) and RNA immunoprecipitation assays were performed to determine the relationship between SNORA73B and its target genes. High‐performance liquid chromatography (HPLC) was performed to detect the pseudouridine content of the mindbomb E3 ubiquitin protein ligase 1 gene (*MIB1*). The stability of *MIB1* mRNA was evaluated using a transcription inhibitor, actinomycin D. By performing co‐immunoprecipitation assays, the change in the ubiquitin levels of the Jagged canonical Notch ligand 1 (Jag 1), caused by SNORA73B and *MIB1*, was identified. RNA‐seq and qRT‐PCR were performed to detect the alternative splicing of the regulator of the chromosome condensation 1 gene (*RCC1*). The TCGA database analysis showed that SNORA73B was highly expressed in EC. SNORA73B promoted cell proliferation, migration, and invasion and inhibited apoptosis. SNORA73B modified the pseudouridine content in *MIB1* and increased the stability of *MIB1* mRNA and protein; thus, it affected Jag 1 ubiquitination and further activated the Notch pathway. SNORA73B also affected the alternative splicing of *RCC1*, increasing the number of transcripts, RCC1‐T2 and RCC1‐T3, which promoted cell proliferation, migration, and invasion. SNORA73B can be a potential target for EC.

## 1 INTRODUCTION

Endometrial cancer (EC) is a common gynaecological malignant tumour that accounts for 2.1% of cancer‐related deaths in females. Many epidemiological studies have confirmed that the incidence of EC is increasing year by year. The average 5‐year survival rate in patients with EC is approximately 80%; however, in patients with advanced distant metastasis, the survival rate is approximately 25%.^1^ The specific pathogenesis of EC is complex and has not been fully elucidated; therefore, it is important to explore the possible mechanisms underlying EC pathogenesis.

Small nucleolar RNAs (snoRNAs), one of the small non‐coding RNAs, are 60–300 nucleotide (nt) in length and mainly exist in the nucleolus.^2^, ^3^ snoRNAs and their core proteins form functional mature granules that guide the posttranscriptional modification and maturation of ribosomal RNAs (rRNAs), small nuclear RNAs (snRNAs), and other cellular RNAs via their antisense guiding elements.^4^, ^5^, ^6^, ^7^ Based on the modification of the target RNAs, there are two types of snoRNAs, box C/D and box H/ACA, which catalyse 2′O‐methylation and pseudouridine modification, respectively.^8^, ^9^ Some studies have shown that some snoRNAs can bind to argonaute 2 (AGO2) to exert microRNA (miRNA)‐like effects.^10^ For example, a study showed that SNORD93‐derived small RNA, sdRNA‐93, bound to AGO2 and targeted the 3′‐untranslated region of the Pipox mRNA in breast cancer and performed efficient gene silencing of the reporter‐gene mRNA, thus functioning like miRNAs.^11^

Recently, studies emerged associating snoRNA dysregulation with cancer progression. For example, the mutation and downregulation of snoRNAU50 are related to prostate and breast cancers.^12^, ^13^ SNORD76 overexpression promotes the tumorigenicity of hepatocellular carcinoma (HCC) via the Wnt/β‐catenin pathway.^14^ Overexpressed SNORA18L5 plays a carcinogenic role in HCC tissues compared with its role in adjacent normal tissues.^15^ According to previous studies, SNORA47, SNORA68, and SNORA78 levels can accurately predict the overall survival rate of patients with lung cancer.^16^, ^17^, ^18^ However, the biological roles of snoRNAs in EC have not been clarified.

In this study, we explored overexpressed snoRNAs in EC tissues using data from The Cancer Genome Atlas (TCGA) and found upregulated SNORA73B in EC tissues. Further experiments confirmed that SNORA73B could promote EC cell proliferation, invasion, migration, and apoptosis inhibition. Additionally, we found that SNORA73B, as a non‐coding RNA transcript of the regulator of the chromosome condensation 1 gene (*RCC1*) intron, could not only target the mindbomb E3 ubiquitin protein ligase 1 gene (*MIB1*) to regulate the Notch pathway but also played an oncogenic role in EC by affecting the alternative splicing of its host gene *RCC1*.

## 2 MATERIALS AND METHODS

### 2.1 Tumour specimens

After obtaining consent from participants, 164 EC specimens and 30 normal endometrium tissue samples were collected from the Department of Gynaecology of the Third Affiliated Hospital of Guangzhou Medical University, Guangzhou, China. The experiments were approved by the ethics committee of the Third Affiliated Hospital of Guangzhou University, Guangzhou, China.

### 2.2 Cell lines and cell culture

Human EC cells (HEC‐1A, HEC‐1B, and Ishikawa) and immortalized endometrial cells (hESC and hEEC) were purchased from the American Type Culture Collection and Jennio Biotech. The hESC cells were cultured with Eagle's Minimum Essential Medium and hEEC cells were cultured with minimum Eagle's medium (MEM). HEC‐1A and HEC‐1B cells were cultured in Dulbecco's modified Eagle's medium (DMEM), whereas Ishikawa cells were cultured in the Roswell Park Memorial Institute‐1640 medium. Cell culture media were supplemented with 10% fetal bovine serum (FBS) and 1% penicillin and streptomycin. According to the standard protocols, all cell lines were cultured and maintained in humidified incubators at 37°C with 5% CO_2_.

### 2.3 Small interfering RNAs, antisense oligo‐nts, and plasmids

Small interfering RNAs (siRNAs) targeting *MIB1* (GGACAAGGATAATACCAAT) and dyskerin pseudouridine synthase 1 gene (*DKC1*) (AGCCTGGATTCGACGGATA), negative control siRNAs, and antisense oligo‐nts (ASOs) to deplete SNORA73B (TGATGTACAGTCCCTTTCCA) were obtained from Ribobio. The synthesized SNORA73B sequence (TCCAACGTGGATACCCTGGGAGGTCACTCTCCCCAGGCTCTGTCCAAGTGGCATAGGGGAGCTTAGGGCTCTGCCCCATGATGTACAGTCCCTTTCCACAACGTTGAAGATGAAGCTGGGCCTCGTGTCTGCGCCTGCATATTCCTACAGCTTCCCAGAGTCCTGTGGACAATGACTGGGGAGACAAACCATGCAGGAAACATAT, Genebank: NR_004404.2) was cloned into the pcDNA3.1 vector (SyngenTech). The constructs were confirmed by sequencing, and the SNORA73B expression was confirmed by quantitative reverse transcription polymerase chain reaction (qRT‐PCR). The pcDNA3.1 plasmids were purchased from Syngentech.

### 2.4 Cell transfection

Cells were transfected with either the above‐mentioned plasmids or 1 μg/mL ASO/siRNA using Lipofectamine 3000 (Thermo Fisher Scientific) for 48 h according to the manufacturer's instructions, following which they were harvested.

### 2.5 RNA isolation and qRT‐PCR assays

The total RNA was extracted using the TRIzol reagent (Takara) and purified using a standard RNA extraction protocol. Complementary DNA (cDNA) was prepared using PrimeScript RT Master Mix (Takara). The relative expression of the mRNA was normalized to that of the reference gene, *GAPDH* (encoding glyceraldehyde‐3‐phosphate dehydrogenase) and/or snRNA, U6. The fold change in the relative expression of mRNAs was calculated using the 2^−ΔΔCt^ method. All experiments were performed in triplicates.

For detecting *RCC1* mRNA variants, a mixture of oligo‐dT (Sigma‐Merck) and different primers were used for amplification. PCR products (Primer 1: amplified RCC1‐T2 [260 bp]. Primer 2: amplified RCC1‐T2 [260 bp], RCC1‐T3 [218 bp], and RCC1‐T1 without exon A or A′ [167 bp]) were separated in a 2% agarose gel by electrophoresis. The band intensity was calculated using ImageJ (NIH).

### 2.6 Western blotting and antibodies

Cells were lysed for 30 min on ice, centrifuged at 4°C for 15 min, and the supernatant was discarded. The total protein in each group was measured using a bicinchoninic acid quantitative kit (Beyotime Biotech Inc). Protein samples were separated using sodium lauryl sulfate‐polyacrylamide gel electrophoresis. The separated proteins were transferred to a polyvinylidene difluoride membrane, incubated with 3% bovine serum albumin at room temperature, and then incubated with primary antibodies. After incubating overnight at 4°C, the membrane was washed five times with tris‐buffered saline–Tween 20 (TBST) every 5 min. After 2‐h incubation with the secondary antibody (1:8000) at room temperature, the membrane was washed three times with TBST every 5 min. Following this, electrochemiluminescence imaging was performed.

The experiment was performed with commercial antibodies, RCC1 (Proteintech, 22142‐1‐AP), MIB1 (Proteintech, 11893‐1‐AP), Notch1 (D6F11, Cell Signalling Technology), Notch2 (Bioss, bs‐21664R), and Notch3 (Proteintech, 55,114‐1‐AP).

### 2.7 Cell viability assay

Transfected HEC‐1B and Ishikawa cells were seeded in 96‐well plates (100 μL/well) at a density of 10^3^ cells/well and cultured for 24 h. Cell viability was calculated based on the absorbance values at 450 nm at 24, 48, and 72 h using Cell Counting Kit‐8 (CCK8, YISEN) according to the manufacturer's instructions. The absorbance of each well at 450 nm was measured using a microtiter plate reader (BioTek). The assay was repeated three times per time point, and the experiment was performed in triplicates.

### 2.8 Flow cytometry apoptosis assay

Cells were seeded at a density of 2 × 10^5^ cells/well in six‐well plates. After 48 h, the cells were harvested and stained with the annexin V‐fluorescein isothiocyanate–propidium iodide apoptosis detection kit (KeyGEN) according to the manufacturer's instructions. The cells were then analysed using flow cytometry (FACS Calibur, BD Biosciences).

### 2.9 Cell migration and invasion assays

Cell migration was determined by wound‐healing assay. Cells were seeded in a six‐well plate at a density of 1 × 10^6^ cells/well (monolayers) and cultured overnight. Cell monolayers in the wells were scratched with a sterile plastic tip, washed two times with phosphate‐buffered saline (PBS), and then cultured in a medium containing 1% FBS. Scratches were imaged using a microscope every 24 h. The area of the cell‐free scratch was determined using ImageJ, and the cell migration index was calculated as the mean of the relative percentage of the covered area compared with that at the 0 h area for each well.

Cell invasion assays were performed in Transwell chambers (8‐μm pore size, Corning Costar Corp.) precoated with Matrigel (Becton Dickinson). Cells were harvested at 48 h post‐transfection and 1 × 10^5^ cells, incubated with serum‐free DMEM, were added to the upper chamber, and those in DMEM containing 10% FBS were added to the lower chamber. After incubation for 48 h before the examination, the cells on the upper surface were wiped with a cotton swab, whereas the cells on the lower surface were fixed in 4% paraformaldehyde for 20 min and stained with 1% crystal violet for 15 min. Finally, the membrane in the chamber was cut and fixed on a glass slide with resin glue. Cells in 10 random fields were counted under a microscope (Olympus).

### 2.10 Xenograft mouse model

Female nude mice (4‐week‐old) were obtained from the Guangdong Laboratory Animal Center (Guangzhou, China) and maintained in an accredited animal facility, according to the proper institutional guidelines. Mice were subcutaneously injected with cells, with stably expressing SNORA73B or a blank vector, in their right flanks. The tumours were monitored continuously, and the tumour volume was calculated as follows:

$$\text{Tumor volume}=4/3\times {\left(\text{width}/2\right)}^{2}\times \left(\text{length}/2\right)$$

All animal experiments were approved by the Ethics Committee of Guangzhou Experimental Animal Center, Guangzhou, China.

### 2.11 Immunohistochemistry

Briefly, fresh tissues were fixed in 10% neutral buffered formalin for 48 h and then embedded in paraffin. The tissue blocks were cut into the desired thickness using a microtome and were affixed on slides. After deparaffinization and rehydration, the tissue sections were blocked and incubated with the *MIB1* (1:200) (11893‐1‐AP, Proteintech) primary antibody at 4°C overnight. The tissue sections were washed with the PBS buffer and incubated with secondary antibodies for 1 h at room temperature. Using a 3,3′‐diaminobenzidine staining kit (DAB150, Sigma‐Aldrich), the sections were stained according to the instructions. The sections were analysed under a phase‐contrast Olympus microscope (Olympus America Inc).

### 2.12 High‐performance liquid chromatography

Instruments manufactured by Waters were used for high‐performance liquid chromatography (HPLC), including the e2695 pump, 2489 UV detector, Empower2 chromatographic workstation, Mettler Toledo 320 pH meter, Mettler Toledo AL10 analytical balance, TLL‐C desktop high‐speed refrigerated centrifuge, and Ecosil C185 μm 4.6 mm × 250 mm chromatographic column. The test drug used was the pseudouridine reference standard (Content 95%, no. AD049131‐19022001, Shanghai Yuhan Chemical Co., Ltd.). Chromatographic pure acetonitrile, redistilled water, and other reagents of analytical grade were selected for this experiment.

For detection, the mobile phase used was a 2.5 mM ammonium acetate (pH = 4.0) buffer containing 5% acetonitrile. The detection wavelength was set at 263 nm, the flow rate was set at 0.8 mL/min, the column temperature was set at 25°C, and 10 μL of samples were injected.

The pseudouridine reference standard was accurately weighed, and double‐distilled water was added to prepare a 2.7 mg/mL solution, which was used as the reference standard stock solution. An appropriate amount of pseudouridine stock solution was accurately sucked and successively diluted with redistilled water to prepare a standard solution containing pseudouridine at 0.8438, 1.6875, 3.3750, 6.750, 13.5000, 27.000, and 54.000 μg/mL concentration, and 10 μL of these solutions were precisely sucked and injected, and their chromatograms were recorded. The chromatographic conditions were observed, and the chromatograms and peak areas (*A*) were recorded. A standard curve was plotted with A on the vertical coordinate and injected concentrations (μg/mL) on the horizontal coordinate. The results showed that the pseudouridine content was in the range of 0.8438–54.0 μg/mL. The *A* value showed a good linear correlation with the injected concentrations, and the regression equation obtained (*R* ^2^ = 1.0) was as follows:

Y=7759.6x−548.57.

A total of 500 μL of samples were centrifuged at 12,000 *g* for 20 min at 4°C, and the supernatants were collected. The samples were assessed under the same chromatographic conditions, and their chromatographs and A were recorded, and finally, the pseudouridine content in the samples was calculated.

### 2.13 RNA sequencing (RNA‐seq)

HEC‐1B cells were transfected with the plasmid containing the SNORA73B fragment or blank vector. Total RNA was isolated with Trizol. RNA samples were prepared with 1 μg of RNA per sample to be used as input material. According to the manufacturer's instructions, sequencing libraries were generated using Neb Next Ultra RNA Library Prep Kit for Illumina (Neb), and an index code was designated to determine the sequences attributed to each sample. Briefly, mRNA was purified, cleaved at an increased temperature, and cDNA was synthesized. The library fragments were purified using the AMPure XP system (Beckman Coulter), and 150–200 bases‐long cDNA fragments were screened. PCR was performed using a polymerase enzyme and universal and index primers. Finally, PCR products were purified (AMPure XP system), and the library was evaluated for quality with the Agilent Bioanalyzer 2100 system.

The index‐coded samples were clustered in cBot Cluster Generation System using T TruSeq PE Cluster Kit v3‐cBot‐HS (Illumia) according to the manufacturer's instructions. Following cluster generation, prepared libraries were sequenced by the Illumina NovaSeq platform, and 150 bp paired‐end readings were generated.

### 2.14 RNA‐binding protein immunoprecipitation assay

Cells were collected and lysed in the RNA‐binding protein immunoprecipitation (RIP) lysis buffer (Beyotime Biotech Inc) along with RNAsin (1000 U/mL) and DNase I (50 U/mL). After centrifugation at 12,000 rpm for 15 min, the supernatant was discarded, and the pellet was incubated with the antiDKC1 antibody (Abcam, ab93777) or immunoglobulin G (Proteintech, B900610) overnight at 4°C. Following this, protein A/G beads (B23202, Bimake) were added, and the mix was further incubated for another 4 h at room temperature. After washing the magnetic beads with the elution buffer, the immunoprecipitated RNA was purified by TRIzol and ethanol and then analysed by qRT‐PCR.

### 2.15 Statistical analysis

All data were analysed statistically using GraphPad Prism 8.0 (GraphPad Inc.) and the Statistical Package for the Social Sciences 20.0 (IBM Corp.) software. Based on the two‐tailed Student's *t*‐test, the difference between the two groups was analysed. Data were presented as the mean ± SD or SE of the mean, and differences at a *p* < 0.05 were considered statistically significant.

## 3 RESULTS

### 3.1 SNORA73B expression is increased in EC

**FIGURE 1:**
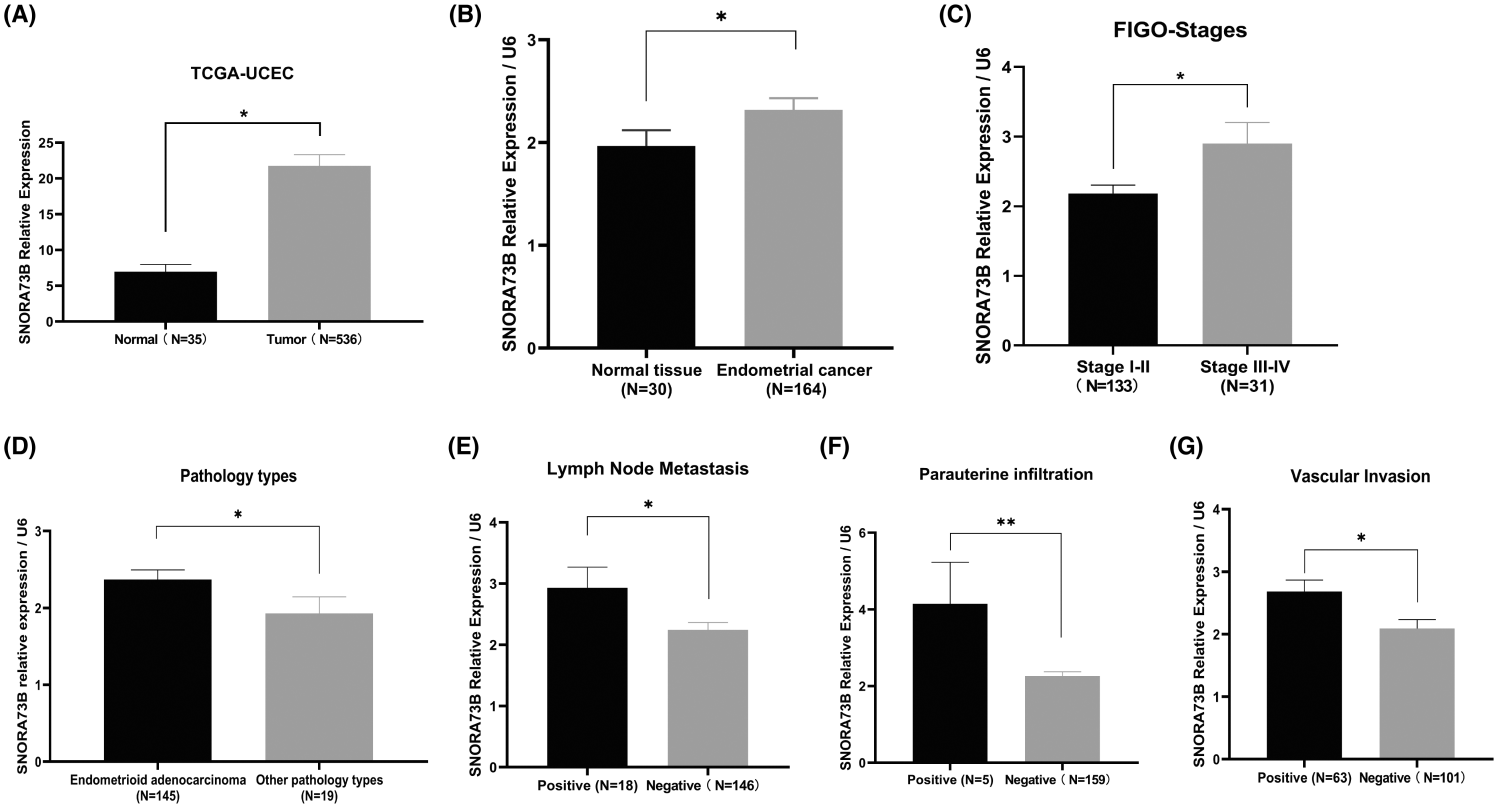
SNORA73B expression is increased in EC. (A) Expression of SNORA73B in uterine corpus endometrial carcinoma cohorts of The Cancer Genome Atlas database. (B) SNORA73B expression in EC tissues (*n* = 164) and normal endometrium tissues (*n* = 30) were detected by quantitative reverse‐transcription polymerase chain reaction. (C) The Federation of Gynaecology and Obstetrics stages: SNORA73B expression at stage III–IV was higher than that at stage I–II. (D) SNORA73B expression was higher in endometrioid adenocarcinoma than in other pathology types. (E) SNORA73B expression was higher in the lymph node metastasis positive group than in the negative group. (F) SNORA73B expression was higher in the para‐uterine infiltration positive group than in the negative group. (G) SNORA73B expression was higher in the vascular invasion positive group than in the negative group. U6 served as a negative control. Data are expressed as the mean ± SD. *p < 0.05, **p < 0.01 (Student's *t*‐test).

The key snoRNAs, with increased expression and involvement in the tumorigenesis and development of EC, were identified using the TCGA–uterine corpus endometrial carcinoma (https://portal.gdc.cancer.gov/projects/TCGA‐UCEC) database. SNORA73B was identified as a potential oncogene of EC (Figure 1A). 164 EC specimens and 30 normal endometrium tissue samples were collected from the Department of Gynaecology of the Third Affiliated Hospital of Guangzhou Medical University. SNORA73B expression in the EC tissues (*n* = 164) and normal endometrium tissues (*n* = 30) was detected by qRT‐PCR and was found to be higher in the EC tissues than in the normal endometrium tissue (Figure 1B) (Table 1). Furthermore, the relationship between SNORA73B expression in the 164 EC tissue samples and their clinical features was analysed. The results showed that SNORA73B expression correlated with the Federation of Gynaecology and Obstetrics (FIGO) stages, lymph node metastasis, para‐uterine infiltration, and the vascular invasion of EC. SNORA73B expression in FIGO stages III–IV was higher than that in stages I–II (Figure 1C). SNORA73B expression in endometrioid adenocarcinoma was higher than in other pathology types (Figure 1D). SNORA73B expression was higher in the lymph node metastasis positive group, vascular invasion positive group, and para‐uterine infiltration positive group than that in the respective negative groups (Figure 1E–G; Table 2). U6 was used as a negative control.

**TABLE 1:** SNORA73B expression in normal endometrial and endometrial carcinoma tissues.

Groups	*N*	SNORA73B expression/U6	*p‐*value
Normal endometrial	30	1.9664 ± 0.1549	***0.004***
Endometrial carcinoma	164	2.3188 ± 0.1142	

*Note*: The fold difference between control samples and endometrial carcinoma was 0.848. Bold and italics value means statistical significance (*p* < 0.005).

**TABLE 2:** Correlation of SNORA73B expression with different clinicopathological features of endometrial carcinoma.

Clinicopathological features	*N*	SNORA73B expression/U6	*p‐*value
Age			
<54	90	2.4585 ± 0.1638	0.09
≥54	74	2.1490 ± 0.1550	
FIGO stages			
I–II	133	2.1837 ± 0.1175	***0.02***
III–IV	31	2.8987 ± 0.3037	
The pathology types			
Endometrioid adenocarcinoma	145	2.3702 ± 0.1255	***0.04***
The other pathology types	19	1.9271 ± 0.2189	
Parauterine infiltration			
Negative	159	2.2615 ± 0.1107	0.08
Positive	5	4.1430 ± 1.0881	
The depth of myometrial invasion			
<1/2	107	2.2208 ± 0.1373	0.13
≥1/2	57	2.5028 ± 0.2033	
Lymph node metastasis			
Negative	146	2.2433 ± 0.1202	***0.03***
Positive	18	2.9311 ± 0.3378	
Pathology classification			
Well	67	2.3334 ± 0.1966	0.46
Moderate‐Poor	97	2.3087 ± 0.1381	
Vascular invasion			
Negative	101	2.0913 ± 0.1414	***0.006***
Positive	63	2.6835 ± 0.1845	

*Note*: Bold and italics value means statistical significance (*p* < 0.05).

Abbreviation: FIGO, The Federation of Gynaecology and Obstetrics.

### 3.2 In vitro and in vivo evaluation of SNORA73B oncogenicity in EC

**FIGURE 2:**
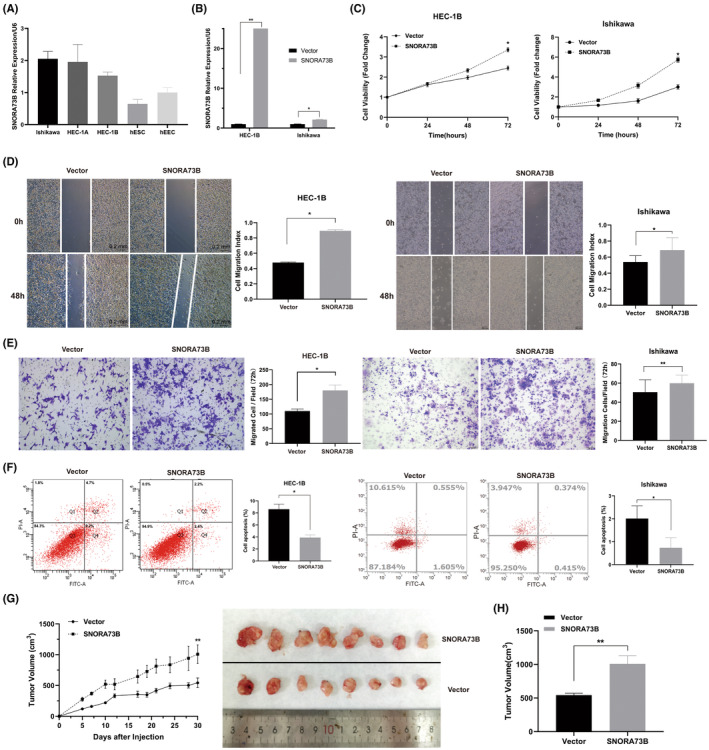
In vitro and in vivo evaluation of SNORA73B oncogenicity in EC. (A) Level of SNORA73B in Ishikawa, human EC cells (HEC)‐1A, and HEC‐1B as determined by quantitative reverse‐transcription polymerase chain reaction (qRT‐PCR) (normalized to that of U6). (B) qRT‐PCR analysis of SNORA73B expression in two cell lines after treatment with an overexpression plasmid vector. (C) Cell proliferation capability detected by cell counting kit‐8 assay. (D) Detection of the migration capability of HEC‐1B and Ishikawa cells transfected with the vector or SNORA73B plasmid by cell scratch assay. (E) Detection of the invasion capability of HEC‐1B and Ishikawa cells transfected with the vector or SNORA73B plasmid by transwell assay. (F) Detection of the apoptosis of HEC‐1B and Ishikawa cells transfected with the vector or SNORA73B plasmid by flow cytometry. (G) Tumour‐bearing mice of different groups were sacrificed and tumours excised from them are shown. The growth curve of the volume of tumours from different groups is shown. (H) Tumour volume of Vector group and SNORA73B group were shown. Data is calculated and shown as the mean ± SD (error bars) from more than three independent repeats. *p < 0.05, **p < 0.01 (Student's *t*‐test).

Next, we evaluated the oncogenic role of SNORA73B in EC cells. SNORA73B expressions were detected in the EC (HEC‐1A, HEC‐1B, and Ishikawa) and the immortalized endometrial (hESC and hEEC) cells (Figure 2A). HEC‐1B and Ishikawa cells that stably expressed SNORA73B were established. (Figure 2B). CCK8 analyses revealed that compared with the cells carrying the blank vector, the proliferation and clone generation of the cells stably expressing SNORA73B were increased (Figure 2C). Transwell and wound‐healing analyses indicated a considerable increase in the mobility and invasion ability of SNORA73B‐overexpressing cells (Figure 2D,E); furthermore, according to the flow cytometry analysis, apoptosis decreased (Figure 2F).

A subcutaneous tumorigenesis model of BALB/c mice was developed by the subcutaneous injection of HEC‐1B cells stably expressing SNORA73B or the blank vector. We measured the longest and shortest diameters of the tumours every 3 days. The relative growth of tumours in the SNORA73B group was considerably higher than that in the vector group (Figure 2G). Additionally, the tumour volume of the SNORA73B group was higher than that of the vector group (Figure 2H). These data indicated that SNORA73B overexpression led to increased cell proliferation, tumorigenicity, invasion, and migration in vitro, and as an oncogene, it induced EC in vivo.

### 3.3 SNORA73B knockdown decreased the proliferation and tumorigenesis of EC

**FIGURE 3:**
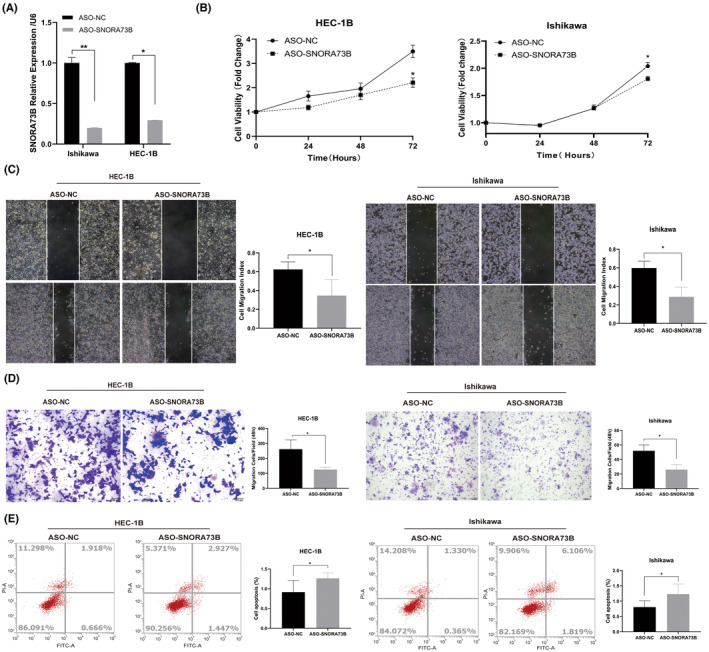
SNORA73B knockdown decreased the proliferation and tumorigenesis of EC. (A) Level of SNORA73B in Ishikawa and HEC‐1B cells transfected with ASO‐SNORA73B as determined by quantitative reverse‐transcription polymerase chain reaction (normalized to that of U6). (B) Proliferation capability of Ishikawa and HEC‐1B cells transfected with ASO‐NC or ASO‐SNORA73B was detected by Cell Counting Kit‐8 assay. (C) Detection of the migration capability of HEC‐1B and Ishikawa cells transfected with ASO‐NC or ASO‐SNORA73B by cell scratch assay. (D) Detection of the invasion capability of HEC‐1B and Ishikawa cells transfected with ASO‐NC or ASO‐SNORA73B by transwell assay. (E) Detection of the apoptosis of HEC‐1B and Ishikawa cells transfected with the vector or SNORA73B plasmid by flow cytometry. Data are calculated and shown as the mean ± SD (error bars) from more than three independent repeats. *p < 0.05, **p < 0.01 (Student's *t*‐test).

To confirm the carcinogenic function of SNORA73B, ASOs were used to detect the effect of SNORA73B knockdown on the oncogene potential of HEC‐1B and Ishikawa cells (Figure 3A). The cell growth was notably inhibited when the SNORA73B knockdown efficiency exceeded 50% (Figure 3B). Transwell and wound‐healing analyses showed that SNORA73B knockdown inhibited the migration and invasion ability of EC cells (Figure 3C,D), and the flow cytometry analysis showed that SNORA73B knockdown induced apoptosis in the cells (Figure 3E). These results indicated that the low expression of SNORA73B might affect the pathogenesis of EC by reducing cell growth, invasion, and migration and increasing apoptosis.

### 3.4 Evaluation of SNORA73B for miRNA‐like function

**FIGURE 4:**
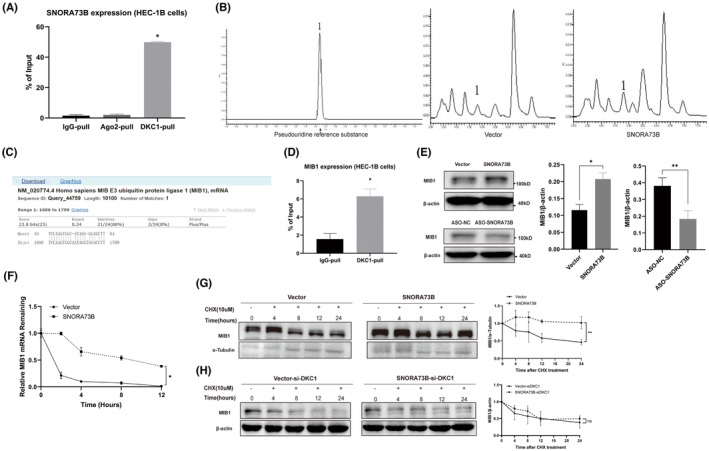
SNORA73B increases the mRNA and protein stability of MIB1 via pseudouridine modification. (A) RNA‐binding protein immunoprecipitation analysis showed that SNORA73B did not bind to argonaute 2 and the binding of SNORA73B and dyskerin pseudouridine synthase 1 (DKC1) was confirmed. (B) High‐performance liquid chromatography showed the content of pseudouridine increased in the SNORA73B overexpression group compared with that in the control group. The peak labelled 1 represents pseudouridine. The detected pseudouridine concentration was calculated based on the standard curve and the area under the peak (the standard curve of the pseudouridine reference substance and area under the peak are not provided). (C) Sequence alignment analysis predicted that the complementary matching gene of SNORA73B was *MIB1*. (D) Mindbomb E3 ubiquitin protein ligase 1 (MIB1) was significantly enriched in the immunoprecipitate of DKC1 compared with that in the control group. (E) Western blotting showed that MIB1 levels increased after SNORA73B overexpression and decreased after SNORA73B knockdown compared with that in the control group. (F) Cells transfected with SNORA73B and the vector were treated with 8 μg/mL actinomycin D for indicated times, then the level of MIB1 mRNA was quantified by quantitative polymerase chain reaction with U6 as a control. (G) After pretreated with the plasmid of the vector and SNORA73B, cells were treated with 4 μM cycloheximide for indicated times. MIB1 levels were detected by western blotting with α‐tubulin as a loading control. (H) After pretreated with the plasmid of the vector or SNORA73B and small interfering RNA targeting DKC1, cells were treated with 4 μM cycloheximide for indicated times. MIB1 levels were detected by western blotting with β‐acin as a loading control. There were at least three samples in each Western‐blot experiment. Data are represented as the mean ± SD from three independent experiments (*p < 0.05).

It has been reported that AGO2 is the core component of RNA‐induced silencing complex (RISC), which not only promotes the degradation of a target mRNA or inhibits its protein translation via the miRNA/siRNA pathway but also regulates miRNA biosynthesis and maturation. Certain nucleolar RNAs can act like miRNAs by combining with AGO2. To determine if SNORA73B can be processed into a smaller RNA fragment with miRNA‐like activity, we used an AGO2 antibody to antagonize AGO2 in cells and detected the RNA attached to AGO2. The result showed that no SNORA73B was bound to AGO2 (Figure 4A); thus, we concluded that SNORA73B did not exhibit a miRNA‐like activity.

### 3.5 SNORA73B increases the *MIB1* mRNA and protein stability via pseudouridine modification

Being a member of box H/ACA snoRNAs, SNORA73B can combine with its target gene via complementary base pairing, inducing *DKC1* to modify the target gene with pseudouridine, thus affecting its protein translation. Initially, SNORA73B and *DKC1* binding was confirmed by performing RIP assay, further revealing that SNORA73B was considerably enriched in the *DKC1* immunoprecipitate group, compared with that in the control group (Figure 4A). Following this, HPLC was performed to detect the change in pseudouridine content. After SNORA73B was transfected into HEC‐1B cells, pseudouridine content increased (Figure 4B). To identify the target gene of SNORA73B, we performed sequence alignment analysis using the Basic Local Alignment Search Tool (BLAST). We identified base complementation of *MIB1* mRNA at 14–16 nt upstream of the ACA cassette in SNORA73B (Figure 4C). The RIP assay confirmed the interaction between *DKC1* and *MIB1*, indicating that *MIB1* was considerably enriched in the *DKC1* immunoprecipitate group compared with that in the control group (Figure 4D). The above results indicated that SNORA73B exhibited its biological role by complexing with *DKC1* and *MIB1*. Western blotting showed that MIB1 levels increased after SNORA73B overexpression compared with that in the control group and decreased after SNORA73B knockdown (Figure 4E). Subsequently, cells transfected with the blank vector or plasmid carrying SNORA73B were treated with the transcription inhibitor actinomycin D (Act.D). SNORA73B expression significantly increased the *MIB1* mRNA stability (*p* < 0.05) (Figure 4F). To assess if the increase in MIB1 levels solely resulted from the increased *MIB1* mRNA stability, the cells were treated with a translation inhibitor, cycloheximide (CHX), which showed that cells overexpressing SNORA73B markedly attenuated MIB1 protein degradation. MIB1 levels were detected by western blotting with α‐tubulin as a loading control. (Figure 4G). After pretreated with the plasmid of the vector or SNORA73B and small interfering RNA targeting DKC1, MIB1 levels were detected by western blotting with β‐acin as a loading control. The protein attenuation delay caused by SNORA73B was counteracted after knocking down DKC1 (Figure 4H). These results indicated that SNORA73B increased MIB1 stabilization in EC cells.

### 3.6 SNORA73B asserts an oncogenic role by targeting *MIB1* via *DKC1* ‐related pseudouridine modification

**FIGURE 5:**
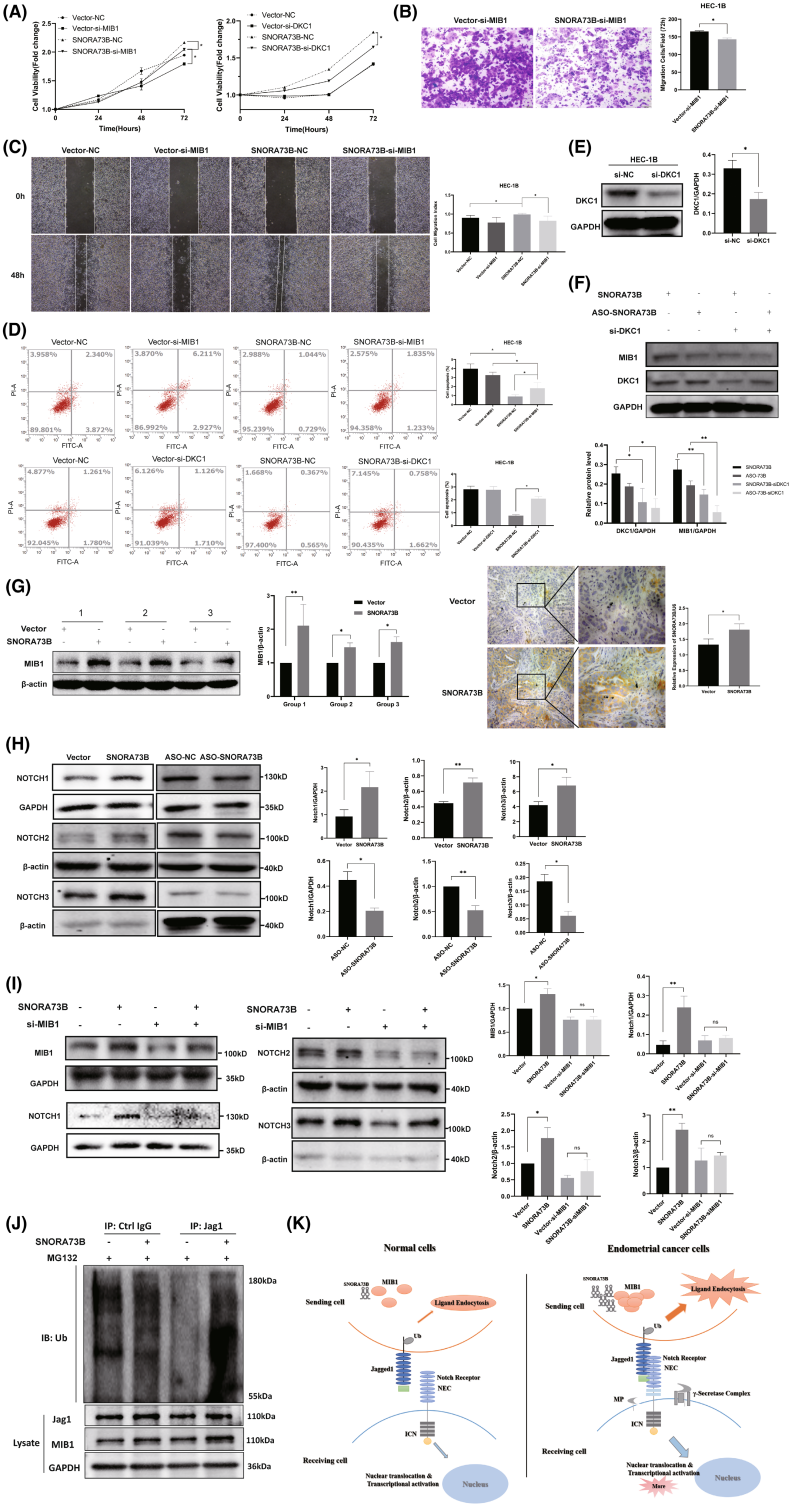
SNORA73B exhibits an oncogenic role by targeting MIB1 via DKC1‐related pseudouridine modification and activates the Notch pathway. (A–D) Downregulation of *MIB1* could partially retain the tumorigenesis function of SNORA73B, such as proliferation, migration, and invasion. *DKC1* knockdown reduces cell proliferation and promotes cell apoptosis. (E) After small interfering RNA for DKC1 (si‐DKC1) transfection, DKC1 levels decreased. (F) Under conditions of *DKC1* knockdown, overexpression or knockdown of SNORA73B did not affect mindbomb E3 ubiquitin protein ligase 1 (MIB1) levels. (G) SNORA73B expression and MIB1 levels were significantly higher in the xenograft tumours of the SNORA73B group than in the vector group. (H) In cells overexpressing SNORA73B, Notch1, Notch2, and Notch3 expression was upregulated. In SNORA73B knockdown cells, Notch1, Notch2, and Notch3 expressions were inhibited. (I) After downregulating *MIB1* expression in SNORA73B high‐expression cells, levels of Notch1, Notch2, and Notch3 showed that the activation of the Notch pathway by SNORA73B was partially offset. (J) Co‐immunoprecipitation results showed that the ubiquitin level of Jag 1 increased because of SNORA73B overexpression. HEC‐1B transfected with SNORA73B plasmids were lysed and subjected to immunoprecipitation with anti‐Jag 1 bead. Input lysates were immunoblotted with antibodies against Jag 1 and MIB1 with glyceraldehyde‐3‐phosphate dehydrogenase (GAPDH) as an internal reference. (K) Schematic diagram of the mechanism of SNORA73B promoting Jag 1 ubiquitination modification and then activating the Notch pathway via MIB1. There were at least three samples in each Western‐blot experiment. Data are shown as the mean ± SD (error bars). *p < 0.05 and **p < 0.01 (Student's *t*‐test).

We transfected cells with control or MIB1‐targeting siRNAs and evaluated if the carcinogenic function of SNORA73B was inhibited by the loss of *MIB1* function in EC. We found that the downregulated MIB1 could partially retain the tumorigenesis function of SNORA73B regarding proliferation, migration, and invasion while promoting apoptosis (Figure 5A–D). We used siRNAs to inhibit *DKC1* expression in order to confirm if the effect of SNORA73B on *MIB1* depended on the *DKC1*‐induced modification of *MIB1*. After siRNA transfection, DKC1 levels decreased (Figure 5E). Under *DKC1*‐knockdown conditions, the overexpression or knockdown of SNORA73B did not affect MIB1 levels (Figure 5F). Downregulated *DKC1* could partially retain the tumorigenesis function of SNORA73B regarding proliferation while promoting apoptosis (Figure 5A,D). The above results indicated that the SNORA73B effect on the MIB1 protein level depends on the catalytic modification of MIB1 mRNA by *DKC1*.

We performed qRT‐PCR, western blotting, and immunohistochemistry (IHC) assays to confirm SNORA73B expression and MIB1 levels in the xenograft tumour tissues. The results showed that SNORA73B expression and MIB1 levels were considerably higher in the xenograft tumour tissues of the SNORA73B group than that of the vector group (Figure 5G).

### 3.7 SNORA73B activates the Notch pathway by promoting the ubiquitination modification of the Jagged canonical Notch ligand 1 (Jag 1) via MIB1

MIB1 is a primary E3 ligase that drives the Notch signal transduction by promoting the ubiquitination of the cytoplasmic tails of Jag 1. This ubiquitination step is critical for in vivo Notch receptor activation; therefore, we investigated if SNORA73B could activate the Notch pathway via MIB1. The RNA‐seq data analysis revealed that SNORA73B overexpression led to the increased expression of several key genes in the Notch pathway, such as Notch1, Notch2, and Notch3 (data not shown). Western blotting confirmed the same results in SNORA73B‐overexpressing HEC‐1B and Ishikawa cells. In SNORA73B‐knockdown cells, the key genes in the Notch pathway, such as Notch1, Notch2, and Notch3, were found to be inhibited (Figure 5H). Then, after reducing *MIB1* expression in SNORA73B high‐expression cells, Notch 1, 2, and 3 levels showed that the activation of the Notch pathway by SNORA73B was partially offset (Figure 5I). The effect of SNORA73B on the ubiquitination level of Jag 1 was investigated by performing co‐immunoprecipitation assays, which showed the increased ubiquitination of Jag 1 when SNORA73B was overexpressed (Figure 5J). The above results indicated that SNORA73B could regulate *MIB1* expression, promoting Jag 1 ubiquitin levels; thus, it could activate the Notch pathway.

### 3.8 SNORA73B affects the alternative splicing of its host gene ( *RCC1* )

**FIGURE 6:**
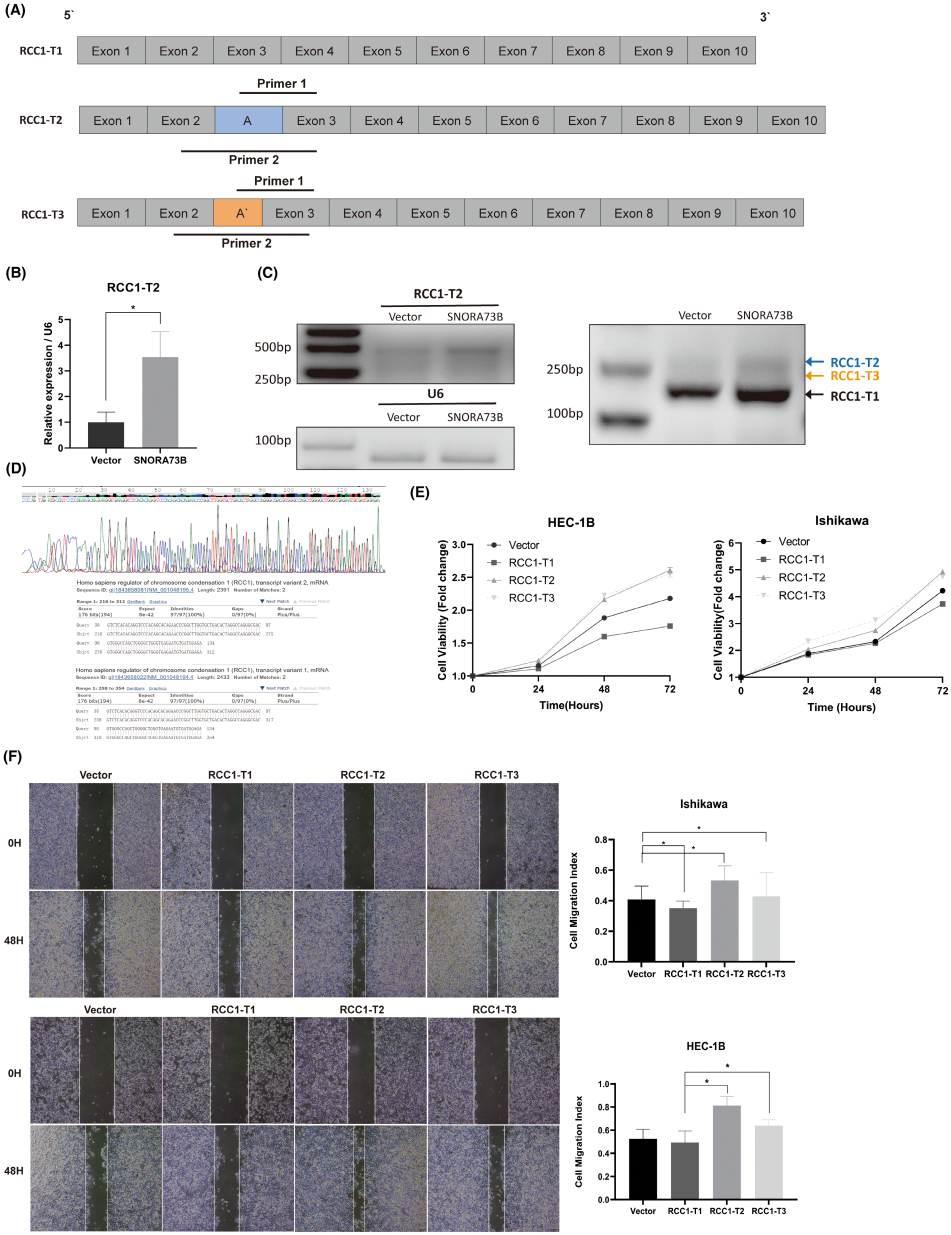
SNORA73B affects the alternative splicing of its host gene RCC1 and RCC1‐T2 and RCC1‐T3 promote the proliferation of endometrial cancer (EC) cells. (A) A series of transcripts of *RCC1* was detected, with transcripts that did not contain the specific exon A being termed RCC1‐T1 and transcripts containing exon A were termed RCC1‐T2 and RCC1‐T3 (RCC1‐T2 and RCC1‐T3 contained exon A of different lengths and the shorter one was named A′). (B) Quantitative reverse‐transcription polymerase chain reaction (qRT‐PCR) of Primer 1 showed that the expression of RCC1‐T2 was upregulated in SNORA73B overexpression cells. (C) PCR with Primer 1 and electrophoresis on 2% agarose gels showed a deeper band in the overexpression group of SNORA73B. This indicated that SNORA73B could promote the expression of RCC1‐T2. PCR with Primer 2 and electrophoresis on 2% agarose gels showed a deeper band around 260 and 218 bp. It showed that transcripts containing Exon A and Exon A′ were upregulated in the SNORA73B overexpression group. (D) The *RCC1* variants of interest were confirmed using Sanger sequencing and BLAST. (E) Overexpression of RCC1‐T2 and RCC1‐T3 in human EC cells HEC‐1B and Ishikawa cells significantly increased cell growth. (F) Overexpression of RCC1‐T2 and RCC1‐T3 in HEC‐1B and Ishikawa cells significantly increased cell migration. Data are calculated and shown as the mean ± SD (error bars) from more than three independent repeats. *p < 0.05 (Student's *t*‐test).

We observed that SNORA73B overexpression could inhibit exon skipping in *RCC1* accorrding to the results of RNA‐seq. Next, we designed a series of primers to detect this alternative splicing in SNORA73B‐overexpressing cells. Various *RCC1* transcripts were detected, with transcripts without the specific exon A being termed RCC1‐T1 and transcripts containing exon A were termed RCC1‐T2 and RCC1‐T3 (RCC1‐T2 and RCC1‐T3 contained exon A of different lengths and the shorter one was named A′; Figure 6A). We performed qRT‐PCR and found that the expression of RCC1‐T2 and RCC1‐T3 increased, whereas RCC1‐T1 expression decreased in SNORA73B‐overexpressing cells by performing qRT‐PCR (Figure 6B) and electrophoresis on 2% agarose gel (Figure 6C,D).

### 3.9 Effects of RCC1‐T1, RCC1‐T2, and RCC1‐T3 on EC

Studies have shown that different transcripts might have different biological functions. We designed plasmids containing these three transcripts and transfected them into cells to test their functions. The assessment of the effect of RCC1‐T1 on the EC cell lines revealed that RCC1‐T1 overexpression in HEC‐1B and Ishikawa cells considerably reduced cell migration and did not promote cell proliferation. In contrast, the overexpression of RCC1‐T2 and RCC1‐T3 in HEC‐1B and Ishikawa cells considerably increased cell growth and migration (Figure 6E,F).

## 4 DISCUSSION

Many processes, such as cell cycle regulation, cell growth, apoptosis, and cell migration, are associated with EC occurrence and development. Previous studies showed that non‐coding RNAs, including miRNAs, long non‐coding RNAs, and circular RNAs, played a crucial role in EC occurrence and development.^6^, ^19^ Several non‐coding snoRNAs closely related to tumour occurrence and development have been recognized over the last two decades. In 2002, a study showed the significantly downregulated expression of a snoRNA, H5sn2, in meningiomas, revealing the abnormal expression of snoRNAs in cancers.^20^ Some researchers have also found that snoRNA is related to the occurrence and progression of other tumours. For example, studies have shown that SNORD89 deteriorates the prognosis of ovarian cancer patients by regulating Notch1‐c‐Myc pathway to promote cell stemness and acts as an oncogene in ovarian tumorigenesis.^21^ However, the role of snoRNAs in EC has not been cleared.

We searched the TCGA database for snoRNA with abnormal expression in the EC. SnoRNAs that can promote the malignant phenotype of EC cells were identified through cell experiments. We have further explored the mechanism of these snoRNAs. For example, we found that SNORD104, as a C/D box snoRNA, binds to 2′‐O‐methylase FBL and induces 2′‐O‐methylation of PARP1 mRNA.^22^ In another study, we found that the combination of SNORD15B and its target gene TRIM25 can up‐regulate the level of TRIM25 and increase its ubiquitination ligase activity, thus blocking the nuclear translocation of p53 in EC and inhibiting the regulation of p53 on downstream genes.^23^ TCGA database exploration and detection of EC samples collected from the Third Affiliated Hospital of Guangzhou Medical University showed that SNORA73B expression was abnormally upregulated in EC. SNORA73B overexpression in HEC‐1B cells and Ishikawa cells indicated that SNORA73B played a major role in cell proliferation, apoptosis, invasion, and migration. Furthermore, a BALB/c mouse model of EC showed that SNORA73B promoted tumour formation in vivo.

Recently, studies have shown that certain snoRNAs can be processed into snoRNA‐like miRNAs with a size of about 18–30 bp, which play a miRNA‐like role, thus silencing downstream target genes.^24^, ^25^, ^26^ In the miRNA/siRNA pathway, AGO2 is the core component of RISC, which promotes target mRNA degradation or inhibits its protein translation and regulates miRNA biosynthesis and maturation. It is located in the central position of the miRNA regulatory pathway.^27^, ^28^ Ender et al. found that the H/ACA cassette could process the snoRNA ACA45 and produce RNA with a length of 20–25 nts, which could combine with AGO to target specific mRNAs.^29^ Xiao et al. recently reported that miR‐605 from the H/ACA cassette plays an important role in stabilizing p53, a tumour‐suppressor‐gene‐encoded protein induced by stress.^30^ These sno‐miRNAs are related to the occurrence of many cancers.^10^ The RIP analysis in the present study showed that SNORA73B did not combine with AGO2, suggesting that SNORA73B did not exhibit its biological function in a miRNA‐like manner.

Based on molecular structural characteristics, snoRNAs are divided into box H/ACA snoRNAs, box C/D snoRNAs, and small Cajal body‐specific RNAs. Among them, box H/ACA snoRNAs can form small nucleolar ribonucleoprotein complexes with pseudouridine synthase DKC1 and three other core proteins, namely NHP2, GAR1, and NOP10 ribonucleoproteins. Under the guidance of snoRNAs, box H/ACA snoRNAs can bind to a target RNA (rRNA, tRNA, and mRNA) via base pairing and catalyse pseudouridine modification at specific sites of the target RNA under the action of pseudouridine synthase DKC1, thereby changing the expression and level of the target mRNA and its encoded protein and participating in multiple physiological and pathological processes.^6^, ^31^ DKC1 is the key pseudouridine synthase, and pseudouridine is its metabolite.^32^, ^33^ The quantitative detection of pseudouridine is important for identifying RNA pseudouridine modification.^34^ Studies showed that pseudouridine modification enhanced the stability of specific mRNAs or affected the efficiency of translation.^35^, ^36^ To verify whether SNORA73B, a member of the box H/ACA snoRNA family, can induce pseudouridine modification, modification sites, and related functions, we performed sequence alignment analysis to predict the base complementary sequence of the SNORA73B sequence and found 21‐nt base complementarity with the *MIB1* sequence at 14–16 nt upstream of the ACA box of SNORA73B. RIP analysis confirmed the binding of SNORA73B and DKC1, *MIB1* and DKC1, whereas HPLC showed a higher pseudouridine level in the high SNORA73B expression group than that in the control group.

To further prove the important role of pseudouridine modification, Act.D and CHX were used, which showed that SNORA73B increased the mRNA and protein stability of MIB1. Subsequently, we used an siRNA to reduce the expression of DKC1, which inhibited the oncogene function of SNORA73B, and SNORA73B overexpression or knockdown affected MIB1 levels in the presence of DKC1 knockdown. These results indicated that SNORA73B combined with *MIB1* via base complementary pairing and modified *MIB1* with a pseudouridine via DKC1, increasing the stability of *MIB1* mRNA, thus resulting in increased protein levels. We speculated that SNORA73B could form complexes with DKC1 and MIB1 to exhibit its biological functions.

Notch signalling initiation requires the intracellular tail ubiquitination of Notch ligands (Delta 1, 3, and 4 and Jag 1 and 2), followed by their endocytosis and activation.^37^, ^38^, ^39^ This process requires two classes of E3 ubiquitin ligases,^40^, ^41^ of which MIB1 is reported to directly interact with and actively regulate all types of Notch ligands via the ubiquitination of Notch receptors.^42^, ^43^, ^44^, ^45^, ^46^ The abnormal Notch pathway has been found in various cancers and is related to the occurrence of tumours, including EC.^47^, ^48^, ^49^, ^50^ Thus, we next assessed the regulatory effect of MIB1 on the Notch pathway in EC. First, we analysed the RNA‐seq data and found increased mRNA levels of *MIB1* and important members of the Notch pathway, such as Notch1, Notch2, and Notch3. The western blot results indicated that after SNORA73B overexpression, the levels of Notch 1, 2, and 3 and MIB1 increased. After the siRNA‐mediated knockdown of *MIB1*, the oncogene effect of SNORA73B was reversed, and the overexpression of SNORA73B did not cause changes in the Notch pathway key genes such as Notch1, Notch2, and Notch3. These results indicated that SNORA73B exhibited its biological function by affecting the Notch pathway by regulating MIB1 levels. Finally, we explored the mechanism of SNORA73B affecting the Notch pathway via MIB1. As previously reported, the change in the ubiquitin level of the Notch pathway ligand Jag 1 caused by MIB1 is critical for in vivo Notch receptor activation.^51^ We detected the ubiquitination of Jag 1 after the overexpression of SNORA73B. As expected, MIB1 levels increased because of SNORA73B overexpression, and the ubiquitination level of Jag 1 in the SNORA73B high expression group was higher than that in the control group. Thus, we confirmed that SNORA73B activated the Notch pathway by promoting the ubiquitination modification of Jag 1 via MIB1.

snoRNAs are involved in a series of biological processes, including alternative splicing.^52^, ^53^ Besides its important role in development, cell differentiation, and the regulation of cell type‐specific functions, alternative splicing is involved in regulating gene expression in cancers.^54^, ^55^ Different transcripts produced by many genes via alternative splicing can be involved in the occurrence and development of tumours by promoting angiogenesis,^56^ inducing cell proliferation,^57^ or inhibiting apoptosis.^58^ For example, the alternative splicing of exon 8 of *VEGFA* is a key determinant of the switch from anti‐angiogenic to angiogenic status.^59^ In 2008, researchers found that p120 catenin, a protein related to tumour invasion ability, exhibited multiple alternative splice isoforms; however, only the full‐length p120 (isomer 1) promoted invasion.^60^ Thus, the ultimate biological function of a gene in tumours is represented by the accumulation of the effects of its different transcripts.

A gene that carries an snoRNA in its intron is called the host gene of the snoRNA.^61^ After gene comparison, we confirmed that SNORA73B was located in the second intron of its host gene, *RCC1*.^62^ RCC1 is an important cell cycle regulator,^63^, ^64^ playing a vital role in nuclear mass transport, mitosis, and nuclear membrane assembly, regulating the occurrence of S‐stage chromosome aggregation, and participating in the regulation of tumour occurrence and development.^65^, ^66^, ^67^ The RNA‐seq results suggested that high SNORA73B expression affected the alternative splicing of diverse genes, including promoting exon 3 retention of its host gene, *RCC1*. Gene comparison showed that exon A retention resulted in the transcription of RCC1‐T2 and RCC1‐T3 (RCC1‐T2 and RCC1‐T3 contain exon A of different lengths and the shorter one is named exon A′). A transcript without exon A retention corresponds to RCC1‐T1. Using a series of primers, we found that SNORA73B overexpression upregulated RCC1‐T2 and RCC1‐T3 expression.

However, the biological functions of these three transcripts are unknown. To further study the biological effects of changes in their expression, the three transcripts were overexpressed, which showed that RCC1‐T2 and RCC1‐T3 promoted the proliferation and migration of EC cells. These results indicated that SNORA73B could increase the content of RCC1‐T2 and RCC1‐T3, promoting tumour proliferation and migration by affecting the alternative splicing of its host gene, *RCC1*, thus acting as an oncogene.

In summary, we found that SNORA73B high expressed in EC tissue from TCGA database and promote the occurrence and development of EC. Then, we used RNA‐seq to evaluate the biological function of SNORA73B in EC and found that SNORA73B could not only affect the expression of a series of tumour‐related genes but also affect the alternative splicing of RCC1. Structurally, SNORA73B belongs to snoRNA H/ACA box, which is one of the main subgroups of snoRNA. SNORA73B can guide DKC1 to target mRNA and cause pseudouridylation of the target mRNA. SNORA73B improved the stability of *MIB1* mRNA and protein by modifying its target gene *MIB1* with pseudouracil, thus affecting the ubiquitination of Jag1 and further activating Notch pathway. SNORA73B also affects the alternative splicing of RCC1, increasing the number of transcripts of RCC1‐T2 and RCC1‐T3, thus promoting cell proliferation and migration. In conclusion, SNORA73B not only modified the target gene by pseudouracil but also affected the alternative splicing of its host gene. The biological function of SNORA73B in EC is the result of multiple mechanisms.

## AUTHOR CONTRIBUTIONS

**Xi Chen:** Data curation (lead); formal analysis (lead); methodology (lead); writing – original draft (lead); writing – review and editing (lead). **Qian‐hui Li:** Investigation (supporting); resources (supporting); software (lead); validation (supporting). **Bumin Xie:** Methodology (supporting); resources (supporting); software (supporting). **Yu‐meng Ji:** Investigation (supporting); resources (supporting); software (supporting). **Yang Han:** Formal analysis (supporting); methodology (supporting); resources (supporting). **Yang Zhao:** Conceptualization (lead); funding acquisition (lead); project administration (lead).

## FUNDING INFORMATION

This work was supported by the National Nature Science Foundation of China, CHN (Nos 82072854; 82272985), the Natural Science Foundation of Guangdong Province (No. 2022A1515012293), the Project for Key Medicine Discipline Construction of Guangzhou Municipality, CHN (No. 2021‐2023‐17), and Science and Technology Projects in Guangzhou (No. 202201020093).

## CONFLICT OF INTEREST STATEMENT

The authors declare no conflict of interest.

## Data Availability

The resources, tools, and codes used in the study are described in the method section of the manuscript. Further data are available from the corresponding author upon request. The public dataset of TCGA analysed during the present study is TCGA‐UCEC, which is available at https://portal.gdc.cancer.gov/projects/TCGA‐UCEC.
